# Auditory-Motor Mapping Training as an Intervention to Facilitate Speech Output in Non-Verbal Children with Autism: A Proof of Concept Study

**DOI:** 10.1371/journal.pone.0025505

**Published:** 2011-09-29

**Authors:** Catherine Y. Wan, Loes Bazen, Rebecca Baars, Amanda Libenson, Lauryn Zipse, Jennifer Zuk, Andrea Norton, Gottfried Schlaug

**Affiliations:** 1 Music and Neuroimaging Laboratory, Department of Neurology, Beth Israel Deaconess Medical Center, Harvard Medical School, Boston, Massachusetts, United States of America; 2 Department of Communication Sciences and Disorders, MGH Institute of Health Professions, Boston, Massachusetts, United States of America; Hôpital Robert Debré, France

## Abstract

Although up to 25% of children with autism are non-verbal, there are very few interventions that can reliably produce significant improvements in speech output. Recently, a novel intervention called Auditory-Motor Mapping Training (AMMT) has been developed, which aims to promote speech production directly by training the association between sounds and articulatory actions using intonation and bimanual motor activities. AMMT capitalizes on the inherent musical strengths of children with autism, and offers activities that they intrinsically enjoy. It also engages and potentially stimulates a network of brain regions that may be dysfunctional in autism. Here, we report an initial efficacy study to provide ‘proof of concept’ for AMMT. Six non-verbal children with autism participated. Prior to treatment, the children had no intelligible words. They each received 40 individual sessions of AMMT 5 times per week, over an 8-week period. Probe assessments were conducted periodically during baseline, therapy, and follow-up sessions. After therapy, all children showed significant improvements in their ability to articulate words and phrases, with generalization to items that were not practiced during therapy sessions. Because these children had no or minimal vocal output prior to treatment, the acquisition of speech sounds and word approximations through AMMT represents a critical step in expressive language development in children with autism.

## Introduction

Communication deficits represent one of the core symptoms of Autism Spectrum Disorders (ASD). Up to 25% of individuals with ASD lack the ability to communicate with others using speech sounds [1]–[2]. While autism is intrinsically a socially isolating disorder, non-verbal children with ASD are further isolated by their severe communication barriers. These children are often taught to use some form of augmentative and alternative communication methods in order to make requests and interact with others [e.g., 3]. Examples of such non-speech approaches include: voice-output communication devices that read messages aloud, manual signs, and the Picture Exchange Communication System (PECS).

The ability to communicate verbally is considered a positive prognostic indicator of outcomes for children with ASD [4]–[5]. Although there are some cases of speech acquisition in older children with ASD, the exact methods used to facilitate this development are often unclear [6]. Few studies have tested the efficacy of a number of interventions for facilitating speech acquisition in non-verbal children with autism using techniques such as orienting cues [1], and other behavioral strategies and prompts [7]. While these preliminary studies showed some improvements in speech production, available interventions that focus specifically on increasing speech output in non-verbal children with ASD remain extremely limited.

The present study evaluated the effectiveness of a novel intonation-based intervention in facilitating speech output in non-verbal children with ASD. This intervention, called Auditory-Motor Mapping Training (AMMT), trains the association between sounds and articulatory actions with the goal of facilitating speech output [8]–[9]. It combines intonation (singing) and the use of a pair of tuned drums to facilitate auditory-motor mapping. The therapist introduces the target words or phrases by simultaneously intoning the words and tapping the drums tuned to the same two pitches. AMMT is conducted through intensive repetition in a highly structured environment, a feature that is common across autism treatments [10].

AMMT has significant therapeutic potential for a number of reasons. First, it capitalizes on the superior musical abilities that have been observed in many children with ASD, and offers activities that they intrinsically enjoy [11]–[13]. This positive response to music and music making may help children with autism engage and interact with others, thus allowing them to participate in activities that could facilitate the acquisition of communication skills. Second, AMMT (which involves intonation and tapping on the tuned drums) engages a network of brain regions that can be activated by visual, auditory, or motor representations of the same actions [e.g.], [ 14–15]. This network involves not only the temporal lobe, but also the posterior inferior and middle frontal regions that overlap with the putative mirror neuron system. Functional MRI studies have shown that frontoparietal motor-related areas are activated not only when individuals are engaged in a motor action, but also when they see or listen to others completing the same action [14]. Mirror neuron dysfunction has been proposed to underlie the communication deficits in ASD [e.g.], [ 16]–[20]. Even if this MNS hypothesis is not supported, a dysfunction of this auditory-motor network may still contribute to the core symptoms of autism. The potential utility of using AMMT to improve verbal output in nonverbal children is reinforced by neuroimaging research showing overlapping and possibly shared neural resources for musical and linguistic stimuli. These overlapping regions also coincide with brain regions that have been identified as the putative mirror neuron network in humans [e.g.], [ 21]–[23]. Because AMMT links the perception of sounds with oral articulatory and motor actions (a process that is critical to meaningful vocal communication), it can engage and possibly strengthen language-related anatomical pathways (such as the arcuate fasciculus and the uncinate fasciculus [24]) that connect auditory and motor brain regions, thereby enabling individuals with ASD to develop their communication skills. Additionally, the communication deficits of children with autism may be due to the oral motor speech deficits observed in language-delayed children with speech apraxia [25], thus further highlighting the possible benefits of incorporating intonation or singing in the AMMT intervention [26]. Finally, a related intonation-based intervention method (Melodic Intonation Therapy) has been shown to be successful in improving speech output in another group of individuals, i.e., stroke patients with nonfluent Broca's aphasia [27]–[29]. This method engages an auditory-motor mapping network as well as sensorimotor feedback regions through the association of hand tapping and intoned vocal output [9], [24], [30].

The use of intonation in facilitating speech development in ASD has been described in two case reports. One documents the language development of a three year-old non-verbal boy, who, after 35 sessions over a 12 month period, was able to combine words and could respond to intoned questions or statements [31]. While the results of this study are encouraging, the lack of a strong methodological design makes it unclear whether the improvement was due to therapy or simply to the boy's delayed language development (i.e., delayed maturation). A more recent case study of a six year-old girl with autism also describes the use of singing in eliciting speech [32]. Progress in this study was largely based on the therapist's impression of the child's vocal production. Nonetheless, these two case studies indicate a particular potential of an intonation-based technique to promote speech production in children who are non-verbal.

The purpose of the present study is to determine the initial efficacy of AMMT in facilitating speech output in non-verbal children with ASD. Given that AMMT is a novel intervention that had not been tested, a single-case design was used to provide “proof of concept” [33]. To determine the therapeutic potential of AMMT, we tested 6 non-verbal children who were beyond the typical age range of initial speech development. All children underwent individual AMMT sessions 5 times per week, over an 8 week period.

## Methods

### Participants

Six non-verbal children between the ages of 5–9 years, with a diagnosis of autism (diagnoses made by pediatric neurologists and neuropsychologists prior to enrollment) participated in the study (see Table 1 for participant characteristics). They were recruited from autism resource centers that service the Greater Boston area. The study was approved by the Institutional Review Board of Beth Israel Deaconess Medical Center. The parents of all children gave written informed consent prior to their participation, and all procedures were conducted according to the approved protocol.

**Table 1:** Participant characteristics.

Child	Gender	Age (yr∶mth)	Diagnosis	Examples of volitional vocal output at baseline	Frequency of speech therapy	Nature of speech therapy
1	M	5∶9	Autism (DSM-IV, CARS)	/ba/, /heh/, /coo/, /leh/	5×/wk at 30 min since age 3	PECS^1^, gestures
2	M	6∶0	Autism (DSM-IV, CARS)	whispered /h/, /k/, /b/	3×/wk at 30 min since age 3	PECS, AAC^2^ device, gestures
3	M	6∶0	Autism (DSM-IV, CARS)	/muh/, /aw/, /p/	2×/wk at 30 min since age 4	PECS, gestures
4	F	6∶3	Autism (DSM-IV, CARS)	/m/, /guh/, /E/	2×/wk at 30 min since age 3.5	PECS, articulation
5	M	6∶9	Autism (DSM-IV, CARS)	/buh/, /guh/, /puh/	4×/wk at 45 min since age 3	PECS, AAC device, video modeling, articulation
6	M	8∶9	Autism (DSM-IV, CARS)	whispered /b/	2×/wk at 30 min since age 3	PECS, signs, gestures

1 PECS = Picture Exchange Communication System.

2 AAC = Augmentative and alternative communication.

We confirmed the participants' diagnoses using the Childhood Autism Rating Scale (CARS) [34]. “Non-verbal” was defined as having the complete absence of intelligible words. All participants had previously received speech therapy for at least 18 months, and demonstrated minimal progress in speech acquisition (i.e., no intelligible words) based on speech-language pathology and parent reports. While receiving AMMT, the participants continued with their regular school programs, but did not engage in any other new treatment schedules. Besides autism, the participants had no major medical conditions such as motor disabilities (e.g., cerebral palsy or tuberous sclerosis), sensory disabilities (e.g., blindness or deafness), and genetic disorders (e.g., Down Syndrome) other than ASD. All participants had receptive language skills of >22 months, based on the Mullen Scales of Early Learning (MSEL) [35], and their absence of intelligible words was confirmed by the Expressive Vocabulary Test [36] and the MSEL. Other inclusion criteria were: the ability to 1) sit in a chair for more than 15 minutes; 2) follow one-step commands without prompting; and 3) imitate simple gross motor and oral motor movements such as clapping their hands, stomping their feet, and opening their mouth.

### Study Design

A single-subject design was employed to provide proof of concept of this new intervention [33]. All participants underwent 40 treatment sessions, conducted 5 days per week over an 8 week period. Each of the individual treatment sessions lasted 45 minutes. For each participant, probe assessment data were collected before, during, and after therapy. Some children required one or more initial familiarization sessions so they became acquainted with the testing room and the therapists. Baseline assessments (each separated by approximately 1 week) were conducted 3 times prior to the start of the intervention. During the treatment period, probe assessments were conducted after sessions 10, 15, 20, 25, 30, 35, and 40. Furthermore, there were two follow-up probe assessments in the post-treatment maintenance phase, spread over an 8-week period (at 4 weeks and 8 weeks), to assess whether changes observed during therapy persisted after treatment ended.

### Intervention

**Figure 1:**
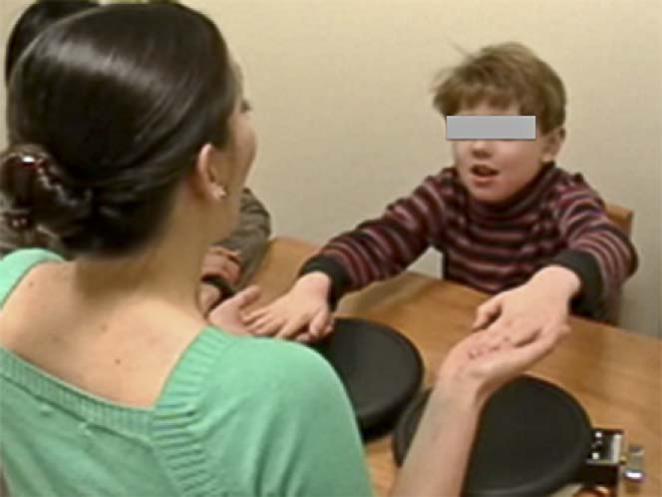
An illustration of an AMMT trial. Therapist guiding a child in the unison production of a target word while tapping the electronic drum pads.

The therapy sessions were conducted in one of the clinical treatment rooms of the Music and Neuroimaging Laboratory at Beth Israel Deaconess Medical Center. During each therapy session, the child was seated facing the therapist (see Fig. 1). A pair of tuned drums was placed between them, with each drum tuned to a fixed pitch (one at C4 or 261.626 Hz, and the other at E^b^ or 311.127 Hz). To establish structure in the treatment environment, each session began with a “Hello Song” and ended with a “Goodbye Song”. The set of 15 items trained during treatment consisted of high-frequency objects, actions, and social words or phrases (e.g., “mommy”, “more please”, “all done”) relevant to the child's activities of daily living. Using Boardmaker pictures (Mayer-Johnson Inc., Solana Beach, CA, 1997) as visual cues, the therapist introduced the target words or phrases by intoning (singing) the words on two pitches, while simultaneously tapping the drums (on the same two pitches), to facilitate bimanual sound-motor mapping. The child was led from listening, to unison production, to partially-supported production, to immediate repetition, and finally to producing the target word/phrase on their own [see 8]. During the treatment sessions, each step could be repeated several times, depending on the child's progress toward mastery of the target. To monitor fidelity of the intervention, all treatment sessions were videotaped. One of the investigators (CW) monitored the therapist's adherence to the protocol, by directly observing a session every week, and also by reviewing 5 other videotaped sessions selected at random. A fidelity monitoring system involving Likert ratings on features of the intervention (e.g., whether the drums were used during the session, whether the therapist practiced only the trained items with the child) was used. All reviewed sessions adhered closely to the AMMT protocol.

### Probe Assessments

During each probe assessment, the child's vocal production was measured in response to two sets of stimuli. One set consisted of 15 trained items (Set 1) and the other contained 15 untrained items (Set 2), and the same items were presented to all children. Both sets of stimuli contained bi-syllabic words or phrases that were matched on: frequency in typical early language acquisition and difficulty of consonants [37]. Set 1 items were practiced with the child during the treatment sessions whereas Set 2 items were not practiced during the sessions, but presented during the probes in a randomized manner. In other words, treatment was applied to Set 1 but not Set 2. The probe procedure was identical to the one outlined in Table 2, but no practice, prompts, or feedback were permitted.

**Table 2:** Structure of an AMMT trial.

Step	Procedure
**1. Listening**	Therapist introduces the target phrase by showing a picture and then intoning (singing) the phrase at a rate of one syllable per second. “More please”.
**2. Unison production**	Therapist and child intone the target phrase together. Therapist intones “Let's sing it together” and in unison with child “more please”.
**3. Partially-supported production**	Therapist and child begin to intone the target phrase together, but halfway through, the therapist fades out while the child continues to sing the rest of the phrase. “More ________”.
**4. Immediate repetition**	Therapist intones and taps the target phrase while the child listens. The child immediately repeats the phrase. “My turn: more please. Your turn: _______”.
**5. Own production**	The child produces the target phase on his/her own one more time. “__________”

To illustrate the steps, the target phrase here is “more please.”

### Speech production measure

The outcome measure of interest was the child's speech production when he or she was presented with the picture stimuli (trained and untrained sets) during the probe assessment sessions. These probe assessments were videotaped and then transcribed offline by independent experienced coders. To minimize experimental bias, coders were blind as to which probe sessions they were coding, and all probes of any one child were transcribed by a single coder in order to maintain consistency in scoring. To examine inter-rater reliability, a subset of the probes (15% of probes across the participants) was transcribed and scored by two coders whose results exhibited high inter-rater reliability (Kappa = 0.71, p<0.001). Furthermore, one coder re-transcribed 20% of her earlier probes to ensure consistency over time, and showed high intra-rater reliability (Kappa = 0.79, p<0.001).

For each target word/phrase, each child's utterances were transcribed and analyzed based on their best production of the target word within a trial, and by determining the number of consonants and vowels produced correctly. The International Phonetic Alphabet was used in the transcriptions to capture variations in speech sounds. Utterances were coded for complexity by examining the accuracy of more complex syllable types such as consonant-vowel (CV) syllables. These structures are commonly examined in the speech of young children [e.g.], [ 38–39]. Because most of the children in this study had minimal speech output prior to treatment, a dependent variable based on approximate CV combinations was considered a reasonable measure of speech production. The criterion for approximate CV production was met if the child produced a consonant approximation combined with a correctly produced vowel. A consonant was considered an approximation when the sound that was produced contained two out of three production dimensions of the target phoneme: voicing (+ and − voice), place (bilabial, labiodental, interdental, alveolar, palatal, velar, glottal) and manner (stop, nasal, fricative, affricate, liquid, glide). For the word “he-llo”, an example of an approximate CV correct would be “he-wo”, and for the phrase “coat on”, an example of an approximate CV correct would be “goa on”. For a child who is completely non-verbal to begin with, an increased ability to approximate words represents a significant and promising step towards speech development [40].

## Results

**Figure 2:**
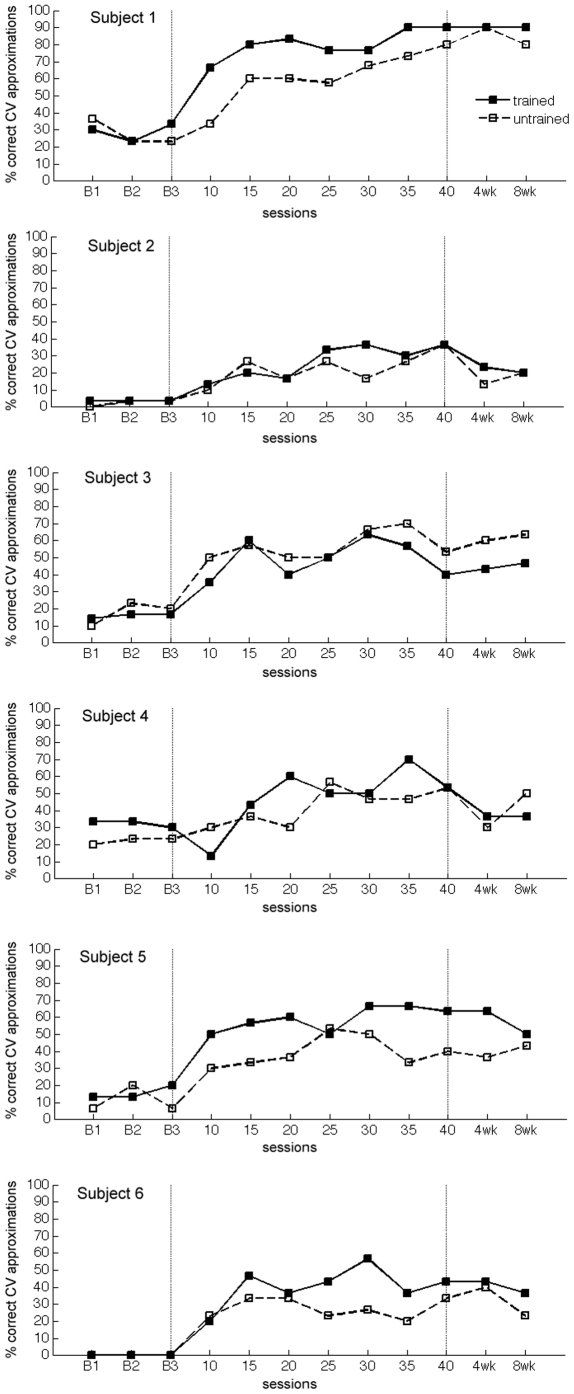
Graphs of each participant's CV production across the baseline, treatment, and follow-up probe sessions.

Fig. 2 shows the percentage of consonant-vowel (CV) approximations produced by each child during the probe assessments administered at baseline, intervention, and follow-up sessions. The x-axis represents the probe assessment sessions and the y-axis represents the percentage of correct CV approximations. Experimental control was attained though the administration of three baseline assessments prior to therapy. As illustrated in Fig 2, all children demonstrated consistently low levels of correct CV approximations prior to treatment. Within 15 sessions of AMMT, all children showed noticeable improvements in speech production. Their improvements gained during treatment were largely maintained in the follow-up sessions. Two-sample paired t-tests comparing CV approximations produced during the best baseline probe versus those made during 40^th^ session revealed that as a group, the children showed improved speech production following therapy (p = 0.001), and this difference was replicated when comparing best baseline probe with that during the follow-up assessments at 4 weeks (p = 0.01) and at 8 weeks (p = 0.003). This indicates that the improvements made during AMMT remained for several weeks after the cessation of daily sessions.

Within each participant, binomial tests of significance were used to determine if the child's speech output during the post-therapy assessment significantly differed from the best pre-therapy baseline session. Based on the expected values obtained from the individual's best baseline, all participants showed significant improvements after therapy. For each individual, we report the proportion correct at the best baseline, and the 95% confidence interval of the proportion correct at session 40. For the trained items, all of the baseline (B) proportions were below the post-therapy 95% confidence intervals (CI): SS (B = 0.17, CI = 0.28 to 0.53), SI (B = 0.33, CI = 0.40 to 0.66), WD (B = 0.03, CI = 0.25 to 0.50), ES (B = 0.13 , CI = 0.50 to 0.75), MZ (B = 0.3 , CI = 0.79 to 0.96), TB (B = 0, CI = 0.30 to 0.57). For the untrained items, all of the baseline proportions were also below the post-therapy 95% confidence intervals (CI): SS (B = 0.23, CI = 0.40 to 0.66), SI (B = 0.23, CI = 0.40 to 0.66), WD (B = 0.03, CI = 0.25 to 0.50), ES (B = 0.2, CI = 0.27 to 0.53), MZ (B = 0.37, CI = 0.68 to 0.89), TB (B = 0, CI = 0.22 to 0.47). The results indicate that AMMT yielded (statistically) significant changes in each child's ability to approximate CV combinations, even for items that were not trained or practiced during the therapy sessions. Table 3 shows the percentage of (exact rather than approximate) words that each child could produce correctly after 40 therapy sessions.

**Table 3:** Percentage of correctly produced target words before and after 40 sessions of AMMT.

Child	Before therapy	After therapy
1	0%	71%
2	0%	8%
3	0%	21%
4	0%	29%
5	0%	21%
6	0%	26%

## Discussion

The purpose of this “proof of concept” study was to determine whether the novel intervention of AMMT could facilitate speech output in non-verbal children with ASD. By using multiple baseline assessments, we were able to compare the vocal production of each individual before treatment to that observed during treatment, and also during the post-treatment follow-ups. These follow-ups allowed us to assess whether any changes observed during therapy persisted outside the daily AMMT routine. On the basis of data collected from 6 children, we suggest that AMMT can be an effective method for helping non-verbal children with autism to increase the range and complexity of their vocal production. Most of the improvements noted at the post-therapy assessment were maintained even 8 weeks after the cessation of the treatment sessions.

All participants underwent 40 treatment sessions, and their rate of progress was greatest within the first 15 sessions. Thus, despite the heterogeneity of autism as a disorder, all participants were able to learn the treatment protocol and to demonstrate improvements in vocal production within a relatively short timeframe. The potential utility of AMMT depends, in part, on whether the increased speech output was restricted to items that were practiced during the therapy sessions. To explore this possibility, we assessed the child's vocal production on a set of untrained items (that were matched on the frequency in typical early language acquisition) during each probe assessment. Our results showed that after therapy, all participants made significant improvements not only in their production of the trained set of items, but also in their production of the untrained set. This indicates that the children successfully learned how to vocalize and produce speech sounds when provided with a model, irrespective of whether the words were specifically practiced during the training sessions.

As demonstrated above, the therapy produced significant improvements in speech production abilities. Participant MZ, in particular, learned to correctly generate several words and phrases (e.g., “all done”, “hello”, “coat on”). Although the speech production abilities of participants remain limited, and they should still be regarded as language delayed, their improvements represent a critical step in the development of expressive language, given that they all had minimal output before treatment. After treatment, all children showed noticeable improvements in the range and complexity of their speech output, as demonstrated by their increased ability to vocalize and produce word approximations. Thus, a realistic outcome of AMMT is significantly improved speech output, rather than the ability to speak and communicate fluently. Because these children had very limited speech output prior to treatment, the acquisition of speech sounds through AMMT is an important gain that provides a foundation for subsequent speech therapy. In other words, the increased repertoire of speech sounds would enable therapists and parents to shape those sounds into words in more functional settings.

The present study is a proof of concept study involving a relatively small number of children, which requires replication and extension. Our results complement those of other pilot studies that teach non-verbal children to speak [e.g.], [ 1,7]. For example, in the study by Koegel et al. [7], an individualized orienting cue (e.g., “high five” gesture, kisses, hugs, novel sounds) was employed to evoke attention prior to verbal prompts. Our intervention did not require such individualized cues, as we administered the same protocol and items to every child. We also implemented follow-up assessments to document the maintenance of treatment benefits. Most importantly, previous studies were conducted on relatively younger children, and therefore, could not rule out the possibility that delayed speech development could account for the improvements. In the study by Rogers et al. [1], the children tested were aged from 2 years to 5 years and 5 months, and in the study by Koegel et al. [7], the children tested were aged from 3 years to 4 years and 8 months. In contrast, the age range in our study was from 5 years and 9 months to 8 years and 9 months at the commencement of therapy. In addition, all children in our study were completely non-verbal, despite having received extensive (at least 2 years) speech therapy before enrollment, it is unlikely that delayed speech development could account for the improvements. Therefore, we can be more confident that the improvements observed in our non-verbal children were due to elements of the AMMT intervention.

While a single subject design is accepted as an appropriate strategy for establishing efficacy of a new intervention in autism [33], and allows for each child to serve as his or her own control, future studies should implement a no-treatment control or alternate treatment group. If the present findings can be replicated in a large-scale study that includes an appropriate control group, then there are a number of potential treatment implications for non-verbal children with autism. One such implication is the possibility of including AMMT in the regular education setting. Current education programs for children with autism often incorporate a didactic, drill-based training [10] that is similar in structure to AMMT. Because the AMMT protocol is relatively straightforward and does not involve the use of expensive equipment, it would not be difficult to integrate this intervention with an established language development curriculum. Ongoing daily sessions of AMMT within the educational setting may not only facilitate the acquisition of speech in otherwise non-verbal children, but also may increase the likelihood of maintaining and building upon their newly-gained speech output as a result of the many opportunities for its functional use in school. Another implication is the expected trajectory of language development in young children diagnosed with autism. Up to 25% of children with autism in preschool years may be non-verbal [41]. If a study shows that AMMT is even more effective in younger children with autism, who are within the critical period for language acquisition, then it may challenge the current expectations of early language interventions in autism. An intervention that can accelerate the rate of speech acquisition in children with autism is likely to improve functional outcomes, and hence quality of life.

Future research could help isolate the fundamental mechanisms underlying effective gains from AMMT. Two main components of the intervention appear to play a role: (1) intonation of words/phases, and (2) motor activities. Intonation (or singing) is known to engage a bilateral network between frontal and temporal regions, which overlaps with language-related pathways such as the arcuate fasciculus and the uncinate fasciculus [24]. It has been argued that a dysfunctional mirror neuron system underlies some of the language deficits in autism [17]. Motor activity (through bimanual tapping the tuned drums) not only captures the child's interest, but also engages or primes the sensori-motor network that controls orofacial and articulatory movements in speech [15], [21], [42]–[44]. The sound produced by the tuned drums may also facilitate the auditory-motor mapping that is critical for meaningful vocal communication [45]–[46].

In summary, early speech development is associated with better outcomes in children with autism [47]–[48]. At present, available interventions that specifically aim to promote speech production in non-verbal children with autism are extremely limited. Novel approaches such as AMMT may facilitate this critical step of language development. Initial efficacy studies such as the one reported here provide “proof-of-concept” and an empirical foundation for future randomized controlled trials.
